# Latitudinal influence on gametogenesis and host–parasite ecology in a marine bivalve model

**DOI:** 10.1002/ece3.7551

**Published:** 2021-05-02

**Authors:** Kate E. Mahony, Sharon A. Lynch, Sian Egerton, Rebecca E. Laffan, Simão Correia, Xavier de Montaudouin, Nathalie Mesmer‐Dudons, Rosa Freitas, Sarah C. Culloty

**Affiliations:** ^1^ School of Biological, Earth and Environmental Sciences University College Cork Cork Ireland; ^2^ Aquaculture and Fisheries Development Centre Environmental Research Institute University College Cork Cork Ireland; ^3^ MaREI Centre for Climate, Energy and Marine Environmental Research Institute University College Cork Cork Ireland; ^4^ Departamento de Biologia and CESAM Universidade de Aveiro Aveiro Portugal; ^5^ UMR 5805 EPOC CNRS Université de Bordeaux Arcachon France

**Keywords:** boom and bust, cockles, fisheries, latitude, parasite–host interactions, reproduction, shellfish, spatial variation, trematodes

## Abstract

Reproduction and parasites have significant impacts on marine animal populations globally. This study aimed to investigate the associative effects of host reproduction and a host–parasite interplay on a marine bivalve, along a geographic gradient of latitude. Cockles *Cerastoderma edule* were sampled from five European sites (54°N to 40°N), between April 2018 and October 2019. A histological survey provided data on trematode (metacercaria and sporocyst life stages), prevalence, and cockle stage of gametogenesis to assess the influence of a latitudinal gradient on both interplays. Sex ratios at the northernmost sites were skewed toward females, and spawning size was reduced at the lower latitudes. Trematode infection did not follow a latitudinal gradient. Localized site‐related drivers, namely seawater temperature, varied spatially, having an impact on cockle–trematode interactions. Spawning was related to elevated temperatures at all sites. Prolonged spawning occurred at southern latitudes, where seawater temperatures were warmer. Trematode prevalence and the impact of trematodes on gametogenesis were found to be spatially variable, but not latitudinally. Therefore, it is not possible to determine the likelihood of boom and bust events in cockles, based on the latitudinal location of a population. In terms of sublethal impacts, it appeared that energy was allocated to reproduction rather than somatic growth in southern populations, with less energy allocated to reproduction in the larger, northern cockles. The demonstrated spatial trend of energy allocation indicates the potential of a temporal trend of reduced cockle growth at northern sites, as a result of warming sea temperatures. This awareness of the spatially varying drivers of populations is crucial considering the potential for these drivers/inhibitors to be exacerbated in a changing marine environment.

## Graphical abstract

A European‐wide field study was conducted on parasitism and reproduction in *Cerastoderma edule*, an ecologically and economically important bivalve. Spatially varying health status of cockles resulted from localized drivers and allocation of energy to reproduction rather than growth at southern locations. Of particular importance was the influence of seawater temperature, with prolonged spawning at southern, warmer sites.

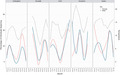

## 1 INTRODUCTION

Boom and bust cycles with a pattern of population growth and decline are a commonly reported phenomenon, particularly in species exploited for fisheries and aquaculture (e.g., Hofmann & Powell, 1998; Korman et al., 2017; You & Hedgecock, 2019). These cycles are particularly evident in marine invertebrates (e.g., Gamboa‐Álvarez et al., 2020; Uthicke et al., 2009; van der Meer et al., 2001), which play vital roles ecologically. The common cockle (*Cerastoderma edule*, Cardiidae) is a suitable model organism due to its wide geographic range and well‐studied biological characteristics (Malham et al., 2012), as well as ecological and commercial significance (Carss et al., 2020). The common cockle is found along Atlantic coasts, from Norway to West Africa (Allcock et al., 1995; Honkoop et al., 2008). This keystone species has previously been shown to experience some biogeographical variation in these cycles on a small scale (Morgan et al., 2013). However, escalations in boom and bust cycles are impacting cockles, with mortality events increasing as a result of factors such as climate change (e.g., extreme temperatures, increased precipitation, variability in water quality) and parasitism (Burdon et al., 2014). Therefore, examining the reproduction of cockles is vital for assessing populations, allowing for appropriate adjustments to be made to the minimum landing size and harvest quota, to provide protection for fisheries and ecosystems into the future.

In bivalves, including cockles, it is evident that many variables impact recruitment spatially and temporally influencing population dynamics and distribution (Beukema et al., 2001; Yankson, 1986). *C. edule* is a dioecious species that can undergo both epidemic and repetitive spawning (Cardoso et al., 2009). Cockles generally exhibit a 1:1 sex ratio (Boyden, 1971), with variations in age/length at first spawning (Cardoso et al., 2009; Elliott et al., 2012; Hancock & Franklin, 1972). The typical reproductive cycle of cockles begins with gametogenesis in spring followed by spawning in the summer (Longshaw & Malham, 2013). Variation is evident in this regime, with cockles in Trondheim, Norway (63°N), spawning for a single month in summer (July) (Rygg, 1970) and cockles in the French Channel (49°N) spawning throughout most of the year (Guillou et al., 1990). Previous multi‐process studies on marine invertebrates have found mesoscale variation in reproduction (Lester et al., 2007). Furthermore, the timing of spawning and gametogenesis differs temporally even in cockles at the same geographic locations (Navarro et al., 1989). Many factors have been proposed as the drivers of spawning and gametogenesis in cockles, including temperature (Gam et al., 2010), water quality (Lusher et al., 2017), immersion time (Honkoop & van der Meer, 1998), and feeding conditions in the previous season (Navarro et al., 1989). A particular cause for concern is the impact of changing climate on cockle reproduction (Morgan et al., 2013). In fact, it has already been shown that extreme events such as hot summers and cold winters negatively impact cockle recruitment and survival (Beukema & Dekker, 2020).

Cockles also serve crucial ecological roles, such as acting as host to a large range of parasites (Longshaw & Malham, 2013), with digenean trematodes being particularly dominant (Thieltges, 2006). As a group of parasites, they have a strong impact on tissue structure and morphology due to their size relative to the host. Trematodes exhibit a complex life cycle and cockles act as a primary (sporocysts) and/or a secondary host (metacercariae) (de Montaudouin et al., 2009), which can have detrimental impacts on their health (de Montaudouin et al., 2012). For example, *Bucephalus minimus* (infecting as a first intermediate host) causes castration (Carballal et al., 2001) and starvation (Dubois et al., 2009). *Gymnophallus choldochus* (first and/or second intermediate host) also causes castration in cockles by eliminating gonad structure (Thieltges, 2006). Parasites are a particularly essential factor to study in host dynamics, because climate change is likely to impact parasite–host interactions, which will not only impact the individual species, but the entire ecosystem due to trophic cascades (Marcogliese, 2008). Furthermore, the expanding northern range of some parasites (e.g., rhizocephalans) may cause local increases in transmission (Gehman et al., 2018) and increasing water temperature may result in trematode infections occurring year‐round (de Montaudouin et al., 2016; Marcogliese, 2001). Such changes in parasite–host dynamics may prove disadvantageous to cockle populations.

The interplay between the reproductive and parasitic processes can have far‐reaching impacts, associated with other spatially and temporally varying drivers. Energy allocation is a fine balance in bivalves, with energy needed for functions including somatic growth, reproduction, and immune response. This energy balance is intertwined with a range of external/environmental factors including temperature, latitude (Clarke, 1987), and parasitism (Lafferty & Kuris, 2009). A common effect of parasites is castration, preventing the reproduction of a host of first intermediate hosts (Lafferty & Kuris, 2009). However, it is difficult to differentiate this from the strategy of a host, diverting energy from reproduction in order to fight the parasite (Hurd, 2001). Host populations experiencing high rates of castration may exhibit altered life histories, with expedited maturity (Lafferty & Kuris, 2009).

This 19‐month study aimed to examine the influence of latitude, as well as the associative influence of trematode infection and site‐specific drivers (environmental and fishing type), on cockle reproductive health and population characteristics across a large proportion of its range. These findings will be vital for understanding this host–parasite system, particularly under the context of climate change.

## 2 MATERIALS AND METHODS

### 2.1 Sample sites

**FIGURE 1:**
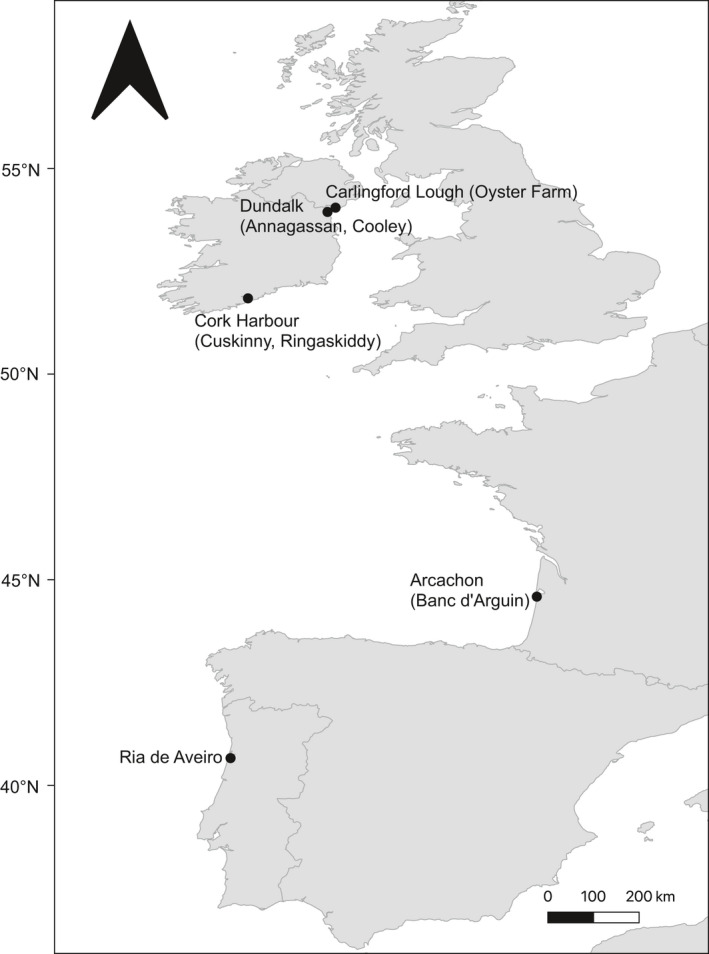
Geographical range of study sites in Ireland, France, and Portugal (bed names indicated in brackets)

Five sites were included in the survey from the northernmost site, Carlingford Lough, to the southernmost, the Ria de Aveiro (Figure 1; Table 1). Only occasional hand harvesting occurs at Carlingford, but the area is important for mussel *Mytilus* spp. and Pacific oyster *Crassostrea gigas* aquaculture (Ferreira et al., 2007). The nearby Dundalk Bay supports a cockle fishery from July to October (Tully and Clarke, 2016). Cockle fishing does not occur at Cork Harbour. Cockle density at Arcachon Bay is very variable (de Montaudouin & Lanceleur, 2011). Furthermore, since 2019, reported densities have been very low. Cockle densities at the Ria de Aveiro are also variable (<10 to ~1,200 individuals per m^2^) due to year‐round harvesting.

**TABLE 1:** Key details of the study sites examined for *Cerastoderma edule*, along with information on individual beds

Site	Bed	Coordinates	Seawater temperature (°C)	Salinity	Abundance
Carlingford Lough (Ireland)	Oyster Farm	54°02′N, 6°10′W	8.2–15^a^	19.8^a^	—
Dundalk Bay (Ireland)	Annagassan	53°52′N, 6°20′W	6–17^a^	33.4–34^a^	1,875 tonnes in 2018^b^
	Cooley	54°00′N, 6°17′W			
Cork Harbour (Ireland)	Cuskinny	51°51′N, 8°15′W	6.9–17.6^a^	33.1–34.8^a^	<10 per m^2^
	Ringaskiddy	51°49′N, 8°18′W			
Arcachon Bay (France)	Banc d'Arguin	44°35′N, 1°14′W	9.5–21.1^c^	32–35^c^	Reaching 2,390 ind/m^c^
Ria de Aveiro (Portugal)	Mira Channel	40°38′N, 8°44′W	11–22^e^	0–36^f^	Reaching 1,200 ind/m^2^

^a^ Copernicus (2020).

^b^ The Marine Institute and Bord Iascaigh Mhara (2018).

^c^ de Montaudouin and Lanceleur (2011).

^d^ Magalhães et al. (2016).

^e^ Vaz et al. (2005).

^f^ Lillebø et al. (2015).

### 2.2 Sample collection

The aim was to collect 30 samples from each bed every other month from April 2018 until October 2019 (19‐month time period). Bimonthly sampling was deemed appropriate, following the slow development of cockles observed in the monthly sampling regime of Morgan et al. (2013). Some deviation occurred due to difficulties locating cockles or where cockle densities were low. At sample sites with rockier substrates (Carlingford and Cork), surfaced cockles were gathered by hand from the intertidal area. This method was deemed more appropriate and time effective with time constraints associated with tidal exchange. At the remaining sites, where sandy and muddy substrates were present, surfaced cockles were gathered by hand in combination with collection of buried cockles using a rake. In total, 1,636 cockles were examined using histology (Table 2). The total number of cockles examined via histology was less than the number collected in the field, due to occasional issues experienced during fixation related to tissue integrity.

**TABLE 2:** Descriptive statistics of all cockles examined in this study and number of males, females, and indeterminate *C. edule* from three Irish sites, one French site, and one Portuguese site

	Male	Female	Indeterminate	Total	Sex Ratio	Chi‐square test	Length (mm)		Growth RINGS	
							Range	Mean	Range	Mean
Carlingford	58	102	69	229	1:1.7	*χ* ^2^ = 12.1, *df* = 1, *p* < 0.001	21.0–45.8	33.8	1–11	4.2
Dundalk	138	178	163	479	1:1.3	*χ* ^2^ = 5.06, *df* = 1, *p* = 0.02	18.1–49.1	32.7	0–9	2.9
Cork	168	177	62	407	1:1.1	*χ* ^2^ = 0.23, *df* = 1, *p* = 0.63	9.5–49.9	32.9	0–13	3.6
Arcachon	100	113	26	239	1:1.1	*χ* ^2^ = 0.80, *df* = 1, *p* = 0.37	16.2–40	28.6	1–7	3.7
Aveiro	123	116	43	282	1:0.9	*χ* ^2^ = 0.21, *df* = 1, *p* = 0.65	7–36	25.8	—	—
Total	587	686	333	1,636	1:1.2	*χ* ^2^ = 7.7, *df* = 1, *p* = 0.006	7–49.9	31.1	0–13	3.4

Environmental data were derived from the Atlantic‐Iberian Bay Irish‐Ocean Physics Analysis and Forecast (Copernicus, 2020). Monthly sea surface temperature data (°C) were derived from this dataset, from coordinates within the vicinity of the sample sites.

### 2.3 Cockle processing

#### 2.3.1 Morphometrics

**FIGURE 2:**
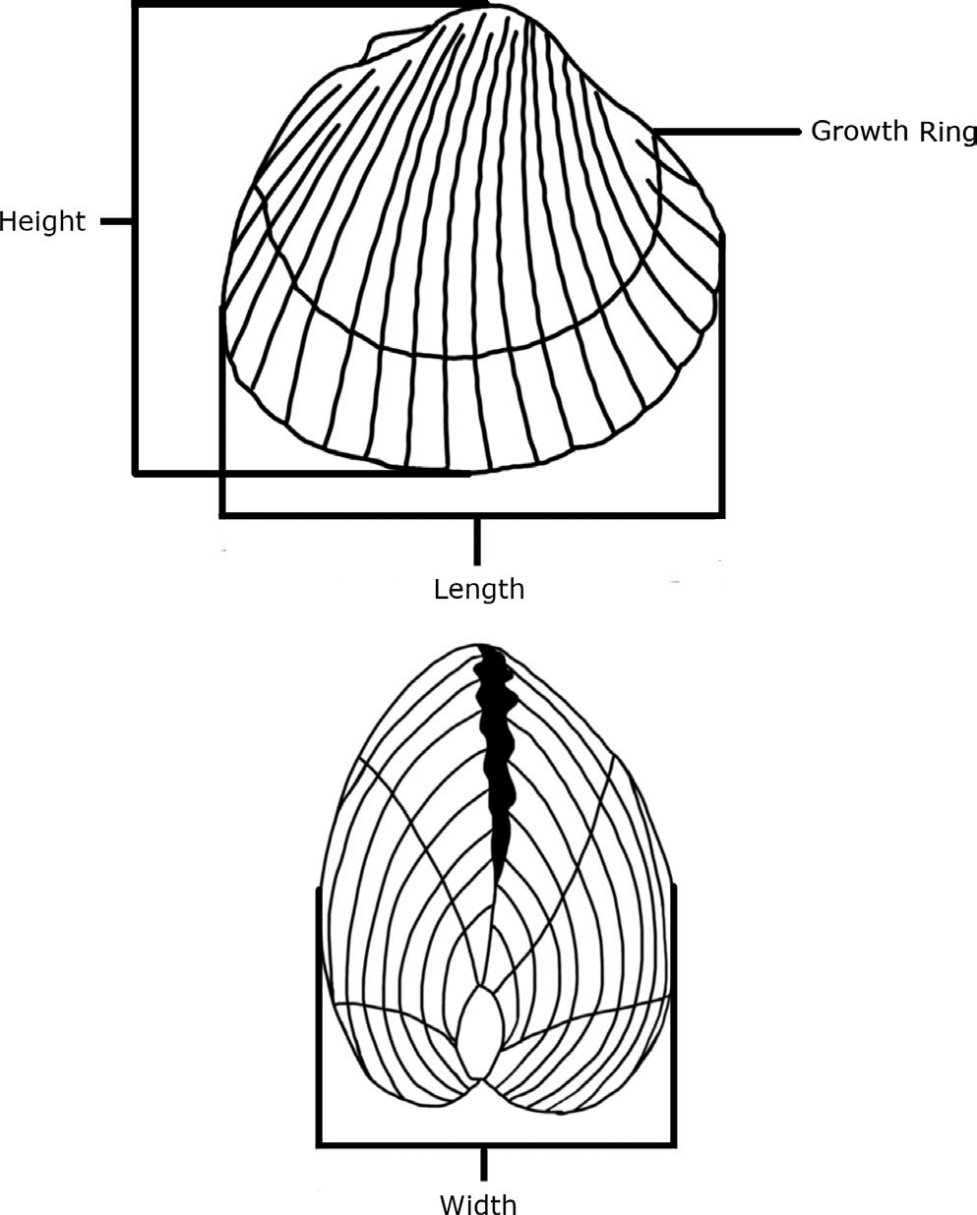
Measurements taken for cockle morphometrics to the nearest mm

Prior to dissection and histology, the whole weight (shell and tissue) and shell morphometrics (Figure 2) were measured for each cockle. Growth rings in cockles are set down each winter (Orton, 1926), although sometimes lines may be hard to distinguish due to warm winters or short cold spells (Ponsero et al., 2009). Therefore, easily distinguishable growth rings were counted to determine an estimation of cockle age at all sites, with the exception of the Ria de Aveiro, where growth rings were not counted.

#### 2.3.2 Histology

The major cockle tissues (mantle, visceral mass, digestive system, foot, and gill) were removed from the shells and fixed in Bouin's solution (Arcachon samples) or Davidson's solution (all other samples) for 24–48 hr (Shaw & Battle, 1957). Samples were then prepared for embedding in wax by running them through a 20‐hr dehydration cycle of graded volumes of ethanol (adapted from Howard et al., 2004). The samples were sectioned to at least 5 μm (3 μm if possible) before hematoxylin and eosin staining (Humason, 1979). Gonad staging was conducted according to the scale described by Morgan et al. (2013), where the gonad was classed according to six stages: early developing, late developing, ripe, spawning/partially spawning, and spent. When one individual exhibited multiple stages or an intermediate between two stages, the dominant stage was assigned. Cockles that did not have identifiable gonad were classed as indeterminate. Presence or absence of trematodes (either sporocysts or metacercaria stages) was also determined (Figure S1.1). This was conducted using a NikonEclipse 80i light microscope, at 4×, 10×, and 40×.

### 2.4 Analysis

The sexual cycle was described at each site and compared with the water temperature. All subsequent analyses were conducted in R (R Core Team, 2019). A regression analysis was conducted to determine if there was a correlation between the percentage of individuals spawning and temperature. Pearson's chi‐squared tests with Yate's continuity corrections were employed to examine the following questions:

1. Did sex ratio differ from 1:1 overall, and at each site/latitude?
2. Did the proportion of trematode infection vary across sites/latitudes?
3. Did the percentage of individuals exhibiting each stage of gametogenesis vary according to trematode infection at the sites/latitudes?

As appropriate, a post hoc analysis comparing the adjusted critical value with the adjusted residuals was conducted to determine which variables were significant at the 5% level. Finally, an analysis of covariance was employed to determine if seawater temperature was associated with percentage of individuals spawning. Assumptions were evaluated using Levene's test for homogeneity of variance and the Shapiro–Wilk normality test.

Data were found to be nonparametric and were therefore submitted to Kruskal–Wallis tests followed by a Dunn test with a Bonferroni correction to examine pairwise differences in spawning length, spawning age, and proportion of indeterminate individuals between sites. Significance level was determined at the 95% probability level. Full statistical results are detailed in Appendix 1.

## 3 RESULTS

### 3.1 Spatial variation in seawater temperature

Seawater temperatures did not follow a strict latitudinal gradient. Mean temperatures at Carlingford ranged from 8.2°C to 13.6°C, with a mean of 11°C. Despite the close proximity of Dundalk with Carlingford, water temperature ranged from 6.5°C to 17.4°C, with a mean of 12.4°C. Water temperature at Cork ranged from 8.5°C to 15.4°C, with a mean of 12.2°C. The mean water temperature at Arcachon Bay was the highest of all of the sites studied (15.6°C) and ranged from 11.1°C to 21.5°C. Despite being the southernmost site, water temperatures were lower at the Ria de Aveiro than in Arcachon Bay and had the narrowest range, from 12.6°C to 16.4°C, with a mean of 14.8°C.

### 3.2 Relationship between sex ratio and indeterminate individuals with latitude

**FIGURE 3:**
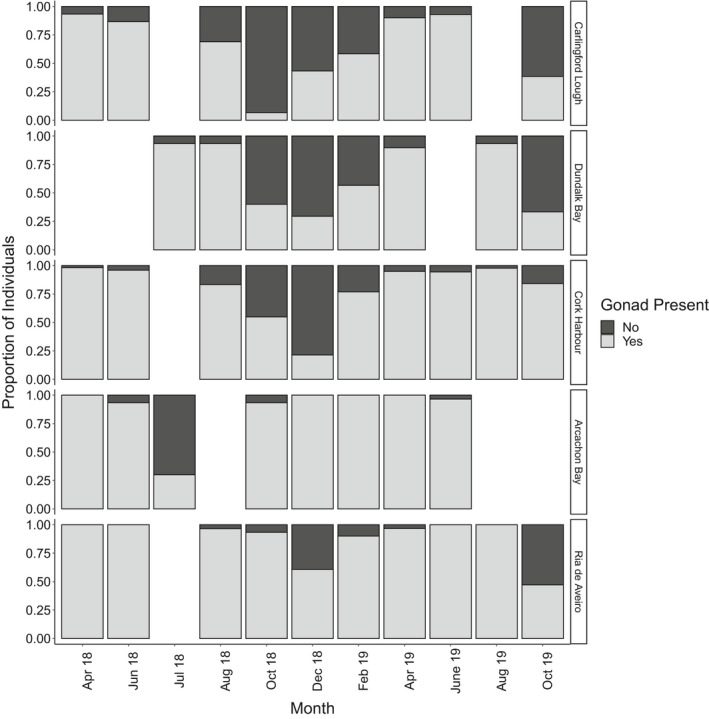
Proportion of *Cerastoderma edule* with and without evident gonad at each site. Months without a column indicate that sampling did not occur

Sex ratio only deviated from the expected 1:1 ratio at the two most northerly sites (Carlingford [1:1.7] and Dundalk [1:1.3]). Indeterminate individuals were found at all sites, with the percentage varying spatially (*χ* ^2^ = 13.7, *df* = 4, *p* = 0.008). Higher percentages of indeterminate individuals were observed at Carlingford (the northernmost site), compared with the Ria de Aveiro (*p* = 0.04‐trend) and Arcachon Bay (*p* = 0.02) (the southernmost sites). Proportions of indeterminate individuals appeared to vary over time, with expected peaks of indeterminate individuals observed during the winter months, while a resting period in gametogenesis occurred (Figure 3). However, the midlatitude site Arcachon Bay, in particular, had a shortened resting period during the summer after massive spawning when the majority of cockles were indeterminate.

### 3.3 Relationship between cockle size, age, and spawning

**FIGURE 4:**
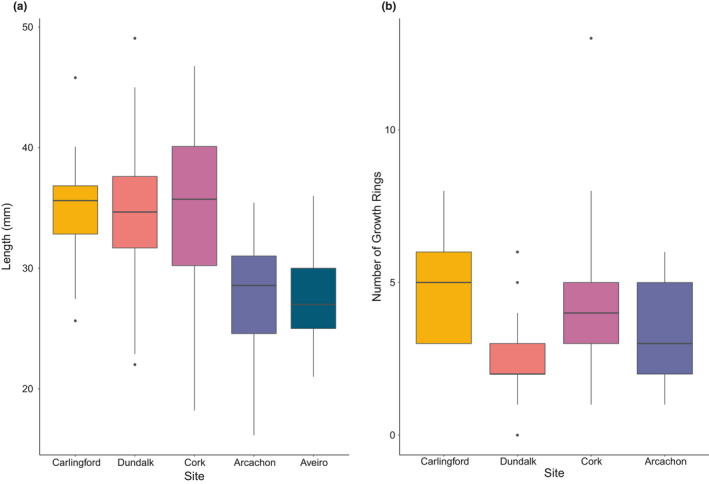
(a) Median (and interquartile range) of length at spawning for cockles at all sites and (b) median (and interquartile range) number of growth rings at spawning for cockles at all sites (excluding Ria de Aveiro)

Cockles spawning at the more southern sites of Arcachon Bay and the Ria de Aveiro were significantly smaller than all of the other sites (*p* < 0.001 in all cases, Figure 4a). However, age did not follow the same gradient, with large variations of spawning age within the Irish sites (Figure 4b).

### 3.4 Sexual cycle and seawater temperature

**FIGURE 5:**
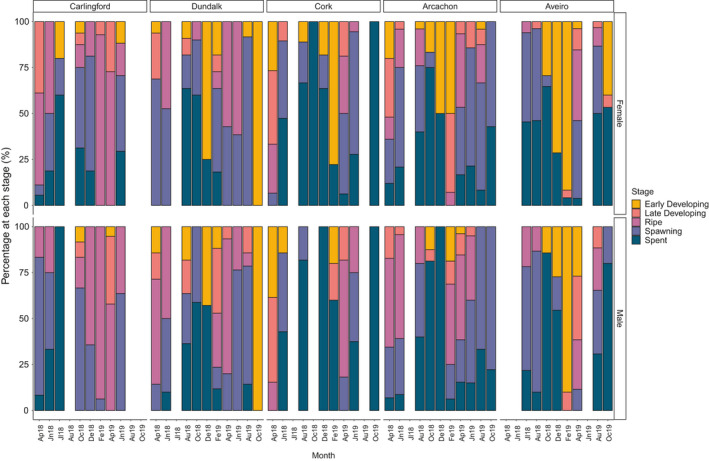
Percentage of *C*. *edule* at each stage of gonadal development at Irish, French, and Portuguese sites. Sampling commenced in April 2018 (Ap18) and was completed in October 2019 (Oc19). Indeterminate individuals were omitted and included in Figure 3. Months with no bar indicate that sampling was not conducted

Spawning duration and frequency varied between Irish sites, between countries and inter‐annually. At Carlingford, a single spawning period (April–October) was observed in both years. In 2018, spawning at Dundalk continued until August in females and October in males, with development recommencing by October 2018 in both sexes. A second, smaller spawning in 2018 was observed in males (20%) in December but did not continue throughout the winter (Figure 5). At Cork, a single spawning event was noted in 2018, ceasing during the winter, indicated by an absence of spent males in February 2019 (Figure 5). However, spawning individuals were observed during all months when sampling took place during 2019. At the southern sites (Arcachon Bay and the Ria de Aveiro), resting periods appeared shorter (Figure 5), with spawning observed during the majority of sampled months (and likely during the interim months due to the numbers of spent individuals observed).

**FIGURE 6:**
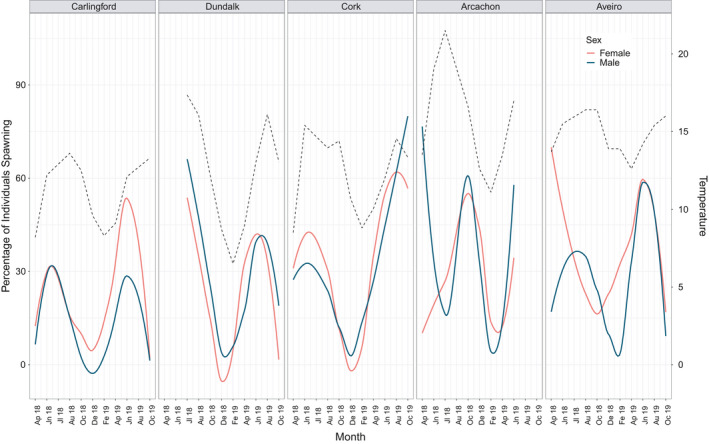
Percentage of males (blue line) and females (red line) spawning during each month of the study, compared with sea water temperature (dotted line), at each site

At the northernmost sites, a synergy between temperature and spawning time was evident (Figure 6). A positive correlation between percentage of individuals spawning and temperature was observed at Dundalk (*p* = 0.002, *F* = 14.2, *df* = 1), and at Cork, there was a similar trend (*p* = 0.061, *F* = 3.994, *df* = 1). No significant correlation was observed at the other sites, but there appeared to be an overall positive trend between increased spawning and increasing temperature (Figure 6).

Differences in the male–female synchronicity of gametogenesis were observed between sites. At Carlingford, spawning generally appeared synchronous between sexes, although more females were spawning in 2019 (Figure 6). At Cork, one of the other northern sites, it appeared that more females were spawning in 2018, with the converse occurring in 2019. At Arcachon Bay, more males were spawning in early 2018 (Figure 6) and at the southernmost site, the Ria de Aveiro, spawning of both sexes was less synchronous in 2018 than in 2019, where similar numbers were spawning in both sexes by June (Figure 6). Spawning at the Ria de Aveiro appeared to be initiated by females in both years (Figure 6).

### 3.5 Patterns of trematode infection and impact on cockle gametogenesis (reproductive cycle)

**FIGURE 7:**
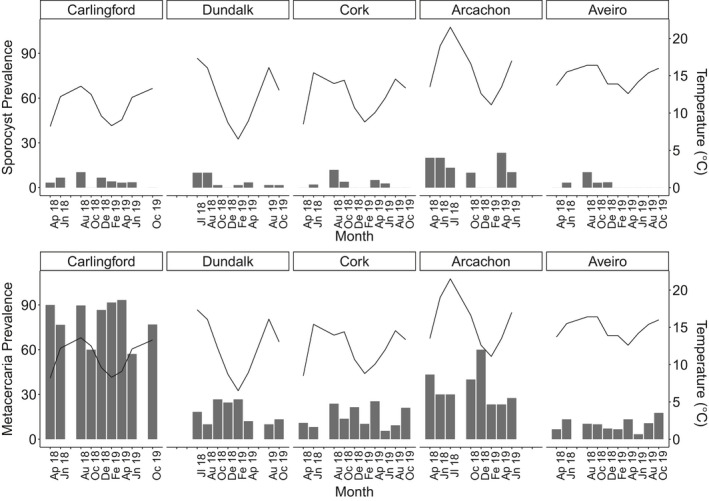
Time series of trematode prevalence (metacercariae and sporocysts) recorded in *C*. *edule* at all study sites, compared with seawater temperature (solid line). Individuals coinfected with metacercariae and sporocysts were included in both graphs. Months without a label indicate that sampling did not occur

Trematode infection varied spatially, with both sporocysts and metacercariae present at all sites but not along a latitudinal gradient. Sporocyst prevalence was highest at Arcachon Bay (12.1%), and metacercariae prevalence was highest at Carlingford (81.7%). However, prevalence of both stages was lowest at the southernmost site (the Ria de Aveiro: sporocysts = 2.1%, metacercariae = 9.6%). Prevalence of infection by metacercariae generally increased with higher water temperature, peaking between ~13.5°C and 17.5°C (Figure 7). However, Arcachon Bay was the exception; therefore, a latitudinal gradient was not obvious, with metacercarial prevalence at Arcachon Bay decreasing gradually over the summer months, when the temperature reached a monthly mean of 22.2°C in July 2019, exceeding that of all other sites, peaking in December at 60% infection (mean temperature at Arcachon = 12.6°C) (Figure 7).

**FIGURE 8:**
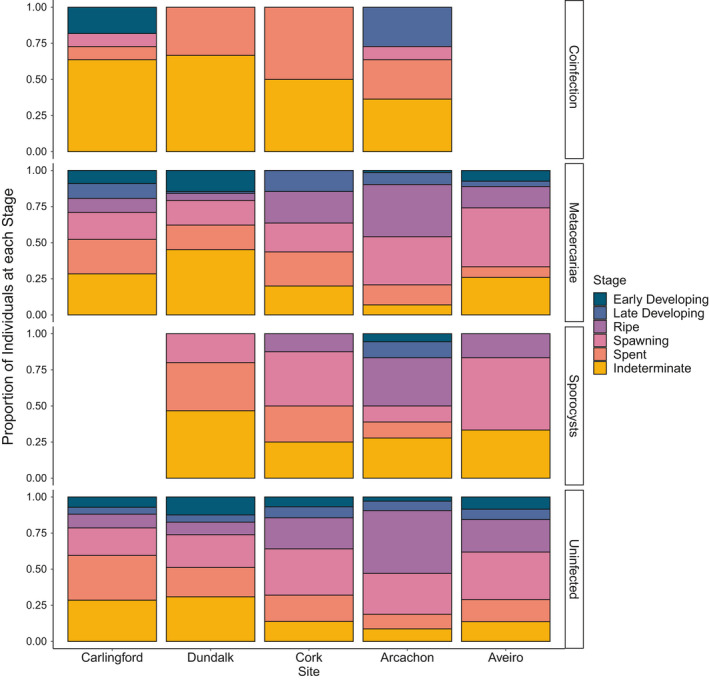
Proportion of *C*. *edule* at each stage of gametogenesis for individuals infected and uninfected by sporocysts and metacercariae. “Coinfection” indicates that an individual was coinfected by sporocysts and metacercariae

Overall, a trend was observed where metacercariae infected individuals were more likely to be indeterminate than to be ripe or spawning (*p* = 0.08, in both cases). Similarly, coinfected individuals were more likely to be indeterminate, rather than ripe or spawning (*p* =.008, in both cases, Figure 8). No significant relationship was observed between cockles solely infected with sporocysts and stage of gametogenesis.

## 4 DISCUSSION

This study highlighted the biogeographical differences in cockle populations, showing that there is an associative influence of trematode infection, temperature, and latitude on population dynamics and reproduction development. Increased water temperatures influenced trematode prevalence, until a threshold temperature was reached (~20°C), when prevalence decreased, potentially impacting cockles at more southern, warmer locations. Trematodes had a deleterious effect on cockle reproduction, with a reduced proportion of spawning individuals and higher numbers of indeterminate individuals among those infected, regardless of latitude or other local factors. While latitude did not have evident impact on the parasitism aspect of the boom and bust cycle, cockle energy allocation and gametogenesis patterns followed a latitudinal gradient, that is, the northernmost sites exhibiting spawning peaks at similar times in the year. Furthermore, spawning occurred almost year‐round at the two southernmost sites, despite variations in average seawater temperature between them. It is also important to note that while bimonthly sampling sufficed at the Irish sites and the Ria de Aveiro (cooler sites), more frequent sampling may be necessary at Arcachon (and possibly other warmer sites), to examine the more accelerated gametogenesis here.

A potential link between sex ratio and latitude was found, with the northernmost latitudes both experiencing skewed sex ratios, despite both varying in terms of fishing pressure and trematode infection. Significantly higher numbers of females were found at these northerly, nearby sites of Carlingford and Dundalk, a phenomenon also seen in other cockle populations (Boyden, 1970; Martínez‐Castro & Vázquez, 2012). A number of explanations may be suggested. First, deviation from a 1:1 sex ratio can result from sex‐specific mortality (Longshaw & Malham, 2013) and it is possible that trematode infection was higher in males, as suggested by a previous study (Morgan et al., 2012). However, further research would be required to test this hypothesis, considering the many species of parasites found in cockles (de Montaudouin et al., 2009; Longshaw & Malham, 2013). Another possibility is that a genetic element of sex determination is at play, with large genetic diversity recorded spatially in cockles previously (Martínez et al., 2015). However, further studies would be required to determine this.

Significant site differences were found in the age and size of spawning cockles. Latitude appeared to be an influencing factor, but fishing activity may also have a role. Older and larger spawning cockles were found at Cork as might be expected as the site is free of any cockle fishing activity. Cockles at Carlingford, a site also free of large‐scale commercial fishing, reached similar ages to Cork cockles. The heavily fished sites had smaller and younger spawning cockles than unfished sites. At the southern sites (the Ria de Aveiro and Arcachon Bay), this may be because cockles allocate more energy toward longer spawning periods, rather than somatic growth. Such variations in reproductive and life history strategies, through differing energy allocation, have been found in other shellfish (Egerton et al., 2020). However, it is also possible that temperature, food availability, and water quality are impacting growth and maturity in cockles (Gosling, 2015); it would be beneficial to further investigate these drivers.

Although seawater temperature, and a range of other local factors, influenced trematode prevalence, it did not follow a latitudinal gradient. Trematode infection levels varied across sites, with the highest prevalence detected at Carlingford. However, it is important to consider that histological methods, such as those used in this study, may result in an underestimation of trematode prevalence, as not all tissue is screened (Morgan et al., 2012). Similar to previous observations (de Montaudouin et al., 2000), at Carlingford there was a high metacercariae prevalence, as well as a high abundance of old cockles. However, metacercariae intensity was not examined and may in fact have been low due to the dilution effect of sympatric *Cr. gigas* (Krakau et al., 2006). Another driver of differences in these sites may be the presence of intermediate and final host species (Byers et al., 2008; de Montaudouin & Lanceleur, 2011; Hechinger & Lafferty, 2005; Thieltges & Reise, 2006, 2007). At Carlingford, birds may not be the factor driving prevalence, due to high human activity on the oyster farm, but it would be worth examining abundance and trematode infection in gastropod species here (Longshaw & Malham, 2013). Interestingly, high numbers of barnacles were observed fouling cockles at Carlingford (Personal Observation). These fouling organisms potentially keep cockle shells from closing entirely, thus increasing the potential for trematode infection, but conversely can predate cercariae (Welsh et al., 2017). Cockle density also differed greatly between Cork and Carlingford, the two sites with low fishing impact. Notably, a lower density of cockles in Cork coincided with lower trematode prevalence. However, it is difficult to ascertain the relationship between host density and trematode prevalence (Magalhães et al., 2017). For example, Cork and the Ria de Aveiro, sites with a large density difference, both reported low trematode prevalence. It may be that these factors are acting additively or synergistically, explaining the differences between sites.

Cockles may go through an over‐wintering stage when their gonad is undifferentiated (Boyden, 1970), and unsurprisingly, high levels of indeterminate individuals were found at all sites, mostly from autumn to winter. However, indeterminate individuals were observed year‐round in the northern sites (Carlingford, Dundalk, and Cork). Many trematode species cause castration (Carballal et al., 2001; Thieltges, 2006), and in this study, a higher proportion of infected cockles were of indeterminate sex, compared to at a ripe or spawning stage. It is generally believed that parasites infecting as sporocysts (reproductive stage) are likely to cause more damage than those infecting as metacercariae (encystment stage) (Wegeberg & Jensen, 1999). However, it was evident that both metacercariae and sporocysts have a deleterious effect on reproduction, with reduced spawning and higher numbers of indeterminate individuals in trematode infected cockle communities.

Findings from this study indicate the geographical variation impacting cockle reproduction, with temperature, trematode infection, and gametogenesis acting associatively. Spawning appeared to be prolonged at the southern sites (the Ria de Aveiro and Arcachon Bay), potentially causing a re‐allocation of energy resources away from individual growth, denoting varying life history strategies among different genetic groups, and sites. Trematode prevalence was related to sea water temperature, while gametogenesis and spawning are impacted by trematodes and water temperature, as shown in this study, as well as by a range of biotic and abiotic factors influencing energy reserves and cockle production (Rueda et al., 2005). The latitudinally varying sizes indicate a link with temporal changes resulting from climate change, with the potential for significant changes in cockle reproduction and host–parasite ecology related to warming seas. These findings are not limited to cockles (Lester et al., 2007), and many of the factors impacting commercially exploited marine species at both a local and regional scale are likely to change as a result of a changing environment, thus highlighting the importance of regular monitoring to follow shifting population dynamics.

## CONFLICTS OF INTEREST

None declared.

## AUTHOR CONTRIBUTIONS

**Kate E. Mahony:** Data curation (lead); formal analysis (lead); investigation (lead); writing – original draft (lead); writing – review and editing (lead). **Sharon A. Lynch:** Conceptualization (equal); methodology (equal); project administration (equal); supervision (equal); writing – review and editing (equal). **Sian Egerton:** Project administration (equal); writing – review and editing (equal). **Rebecca E. Laffan:** Data curation (equal); writing – review and editing (equal). **Simão Correia:** Data curation (equal); investigation (equal); writing – review and editing (equal). **Xavier de Montaudouin:** Data curation (equal); methodology (equal); writing – review and editing (equal). **Nathalie Mesmer‐Dudons:** Data curation (equal); writing – review and editing (equal). **Rosa Freitas:** Data curation (equal); writing – review and editing (equal). **Sarah C. Culloty:** Conceptualization (equal); funding acquisition (equal); methodology (equal); project administration (equal); supervision (equal); writing – review and editing (equal).

## Supporting information

Supplementary Material

## Data Availability

Data used in this study is available in the Dryad Digital Depository https://doi.org/10.5061/dryad.s1rn8pk7b
