# Supplementary material for: Latitudinal influence on gametogenesis and host–parasite ecology in a marine bivalve model
Source: Ecol Evol. 2021 May 2;11(11):7029–41. doi: 10.1002/ece3.7551 (PMC8207143; doi:10.1002/ece3.7551)
Supplement: Supplementary file 1 — Supplementary Material [file ECE3-11-7029-s001.docx]

Supplementary Material

| 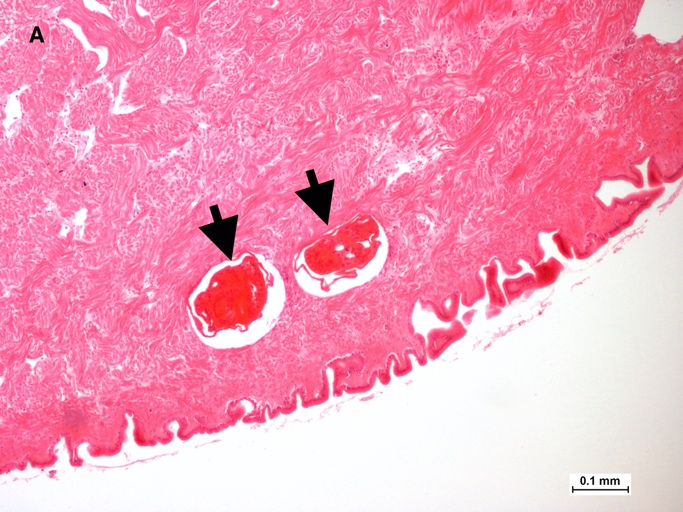 | 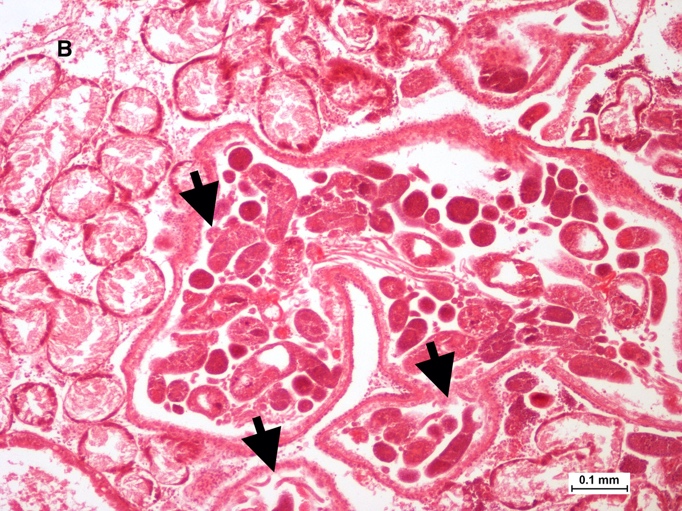 |
| --- | --- |

Figure S1.1. Representative images of (A) trematode metacercariae located in the foot of *Cerastoderma edule* and (B) trematode sporocysts located in cockle digestive tissue tissue. Arrows point to the respective trematode stages.

Table S1.1. Numbers of cockles examined by histology, for each bed and month.

|  | **Arcachon** | **Aveiro** | **Carlingford** | **Dundalk Bay** | | **Cork Harbour** | |
| --- | --- | --- | --- | --- | --- | --- | --- |
|  | Arguin | Aveiro | Oyster Farm | Annagassan | Cooley | Ringaskiddy | Cuskinny |
| Apr-18 | 30 | 30 | 30 | 0 | 0 | 30 | 25 |
| Jun-18 | 30 | 30 | 30 | 0 | 0 | 30 | 19 |
| Jul-18 | 30 | 0 | 0 | 30 | 30 | 0 | 0 |
| Aug-18 | 0 | 29 | 29 | 30 | 30 | 28 | 14 |
| Oct-18 | 30 | 30 | 15 | 30 | 30 | 28 | 23 |
| Dec-18 | 30 | 28 | 30 | 31 | 30 | 5 | 9 |
| Feb-19 | 30 | 30 | 24 | 30 | 30 | 24 | 15 |
| Apr-19 | 30 | 30 | 30 | 29 | 29 | 30 | 29 |
| Jun-19 | 29 | 30 | 28 | 0 | 0 | 28 | 8 |
| Aug-19 | 0 | 28 | 0 | 30 | 30 | 27 | 16 |
| Oct-19 | 0 | 17 | 13 | 30 | 30 | 10 | 9 |

Table S1.2. Result of Dunn Tests with Bonferroni correction to determine if the percentage of indeterminate individuals differed between sites, H_0_ rejected when p<0.025.

|  | Arcachon | Aveiro | Carlingford | Cork |
| --- | --- | --- | --- | --- |
| Aveiro | 0.999 | - |  |  |
| Carlingford | **0.026** | 0.036 | - | - |
| Cork | 0.661 | 0.939 | 0.801 | - |
| Dundalk | 0.056 | 0.080 | 0.999 | 0.999 |

Table S1.3. Result of Dunn Tests with Bonferroni correction to determine if mean length at spawning differed between sites, H_0_ rejected when p<0.025.

|  | Arcachon | Aveiro | Carlingford | Cork |
| --- | --- | --- | --- | --- |
| Aveiro | 0.999 | - |  |  |
| Carlingford | **<0.001** | **<0.001** | - | - |
| Cork | **<0.001** | **<0.001** | 0.999 | - |
| Dundalk | **<0.001** | **<0.001** | 0.999 | 0.999 |

Table S1.4. Results of Dunn Tests with Bonferroni correction to determine if number of growth rings of spawning individuals differed across sites, H_0_ rejected when p<0.025.

|  | Arcachon | Carlingford | Cork |
| --- | --- | --- | --- |
| Carlingford | **0.003** | - | - |
| Cork | 0.488 | **0.021** | - |
| Dundalk | **0.003** | **<0.001** | **<0.001** |

Table S1.5. Results of a chi square test examining if the proportions of metacercariae infected individuals varied across sites

| Comparison | Adjusted Chi Square p value |
| --- | --- |
| Arcachon vs Aveiro | **<0.001** |
| Arcachon vs Carlingford | **<0.001** |
| Arcachon vs Cork | **<0.001** |
| Arcachon vs Dundalk | **<0.001** |
| Aveiro vs Carlingford | **<0.001** |
| Aveiro vs Cork | **<0.001** |
| Aveiro vs Dundalk | **<0.001** |
| Carlingford vs Cork | **<0.001** |
| Carlingford vs Dundalk | **<0.001** |
| Cork vs Dundalk | **<0.001** |

Table S1.6. Results of chi square test examining if the proportions of metacercariae infected individuals differed at each stage of gametogenesis.

| Comparison | Adjusted Chi Square p value |
| --- | --- |
| Indeterminate vs Early Developing | 0.3640 |
| Indeterminate vs Late Developing | 0.9999 |
| Indeterminate vs Ripe | 0.0802 |
| Indeterminate vs Spawning | 0.0802 |
| Indeterminate vs Spent | 0.6350 |
| Early Developing vs Late Developing | 0.5760 |
| Early Developing vs Ripe | 0.8550 |
| Early Developing vs Spawning | 0.8550 |
| Early Developing vs Spent | 0.7940 |
| Late Developing vs Ripe | 0.2940 |
| Late Developing vs Spawning | 0.2940 |
| Late Developing vs Spent | 0.8220 |
| Ripe vs Spawning | 0.9999 |
| Ripe vs Spent | 0.3120 |
| Spawning vs Spent | 0.3120 |

Table S1.7. Results of chi square test examining if the proportions of metacercariae and sporocyst coinfected individuals differed at each stage of gametogenesis.

| Comparison | Adjusted Chi Square p value |
| --- | --- |
| Indeterminate vs Early Developing | 0.4680 |
| Indeterminate vs Late Developing | 0.8280 |
| Indeterminate vs Ripe | **0.0077** |
| Indeterminate vs Spawning | **0.0077** |
| Indeterminate vs Spent | 0.4680 |
| Early Developing vs Late Developing | 0.9220 |
| Early Developing vs Ripe | 0.3730 |
| Early Developing vs Spawning | 0.7600 |
| Early Developing vs Spent | 0.9200 |
| Late Developing vs Ripe | 0.1120 |
| Late Developing vs Spawning | 0.2650 |
| Late Developing vs Spent | 0.9999 |
| Ripe vs Spawning | 0.8280 |
| Ripe vs Spent | 0.1120 |
| Spawning vs Spent | 0.1710 |

Table S1.8. Mean length (mm) and number of growth rings for cockles infected with trematodes at all study sites, ± 1 standard deviation. Growth ring data absent for the Ria de Aveiro. All cockles refers to both infected and uninfected individuals.

| Site | Metacercariae | | Sporocysts | | All Cockles | |
| --- | --- | --- | --- | --- | --- | --- |
|  | Length | Growth Rings | Length | Growth Rings | Length | Growth Rings |
| Carlingford Lough | 33.7 ± 3.9 | 4.2 ± 1.6 | 33.6 ± 4.0 | 4.1 ± 2.3 | 33.8 ± 3.9 | 4.2 ± 1.6 |
| Dundalk Bay | 33.5 ± 5.8 | 3.4 ± 1.6 | 35.1 ± 5.2 | 3.1 ± 1.1 | 32.7 ± 5.7 | 2.9 ± 1.5 |
| Cork Harbour | 35.5 ± 7.8 | 4.2 ± 2.0 | 36.9 ± 4.0 | 3.3 ± 1.1 | 32.9 ±8.4 | 3.6 ± 1.8 |
| Arcachon Bay | 29.4 ± 4.3 | 3.9 ± 1.2 | 30.3 ± 4.1 | 3.7 ± 1.5 | 28.6 ±4.5 | 3.7 ± 1.4 |
| Ria de Aveiro | 26.2 ± 6.9 | - | 29.3 ± 3.6 | - | 25.8 ±5.3 | - |
